# Refractive surgery in glaucoma and suspected glaucoma patients: Critical analysis of the literature and clinical recommendations

**DOI:** 10.5935/0004-2749.2024-0351

**Published:** 2025-06-24

**Authors:** Sarah Mariz Veras Pinto Figueiredo, Olívia Moura de Paula Ricardo, Wilma Lelis Barbosa, Tiago Santos Prata

**Affiliations:** 1 Department of Ophthalmology and Visual Science, Universidade Federal de São Paulo, São Paulo, SP, Brazil; 2 Glaucoma Institute, Belo Horizonte, MG, Brazil; 3 Department of Ophthalmology, Universidade de São Paulo, São Paulo, SP, Brazil; 4 Department of Ophthalmology, Mayo Clinic, Jacksonville, FL, USA

**Keywords:** Glaucoma, Intraocular pressure, Myopia, Refractive surgery

## Abstract

Myopia is a significant risk factor for glaucoma and a growing public health problem worldwide. Detecting glaucomatous changes in highly myopic eyes is diagnostically challenging due to the abnormal appearance of the optic nerve head. These patients also have a greater biomechanical susceptibility to pressure-induced glaucomatous damage. Refractive surgery has become increasingly popular, and many candidates for refractive surgery are myopic. Therefore, we sought to review the aspects of patient evaluation in those who have undergone refractive surgery for myopia concerned with the detection and monitoring of glaucoma development. We identified several important elements of patient evaluation for glaucoma after refractive surgery. These included the need for both structural and functional assessments before and after surgery, and the importance of monitoring for postoperative biomechanical changes in the cornea and their impact on intraocular pressure. We conclude that, in patients who undergo refractive surgery for myopia, it is essential to assess for the presence of glaucoma, to identify staging, and to plan for long-term control of the disease, regardless of IOP.

## INTRODUCTION

Refractive surgery has evolved over the years. It has proven to be a very safe procedure that produces excellent visual results for patients with low to moderate ametropias. The number and efficacy of refractive surgery techniques have grown and are widely used to treat a variety of refractive errors in different corneal patterns^(1)^.

Glaucoma is the leading cause of irreversible blindness worldwide, and open-angle glaucoma (OAG) is the most prevalent type. Myopia is a well-known risk factor for OAG^(2)^. Patients undergoing refractive surgery often have moderate to high myopia, which is associated with a higher predisposition to glaucoma development^(3)^. Such patients are often young, when the prevalence of glaucoma is low, so diagnosis can be challenging. Intraocular pressure (IOP), the main risk factor for the disease, varies a great deal significantly in this population. In addition, the appearance of the optic nerve head, which is crucial for the proper investigation of glaucomatous damage, can have significant anatomical variations in myopic eyes^(4)^.

Given the increasing popularity of corneal refractive surgery, combined with the shift toward aging populations, the prevalence of patients diagnosed with glaucoma who have had prior refractive surgery will likely rise. This literature review aims to consider the aspects involved in evaluating and diagnosing glaucoma development in patients who have previously undergone refractive surgery. We also aim to consider the ongoing monitoring of the disease.

## MYOPIA AND GLAUCOMA

The myopia epidemic is a growing public health problem, and its prevalence is expected to increase to 5 billion people (half of the world’s population) by 2050. This will have a significant social impact due to the uncorrected refractive errors and irreversible vision loss caused by myopic macular degeneration and glaucoma^(3)^. Myopia is a well-established risk factor for glaucoma, and the risk increases with age. A study by Pan et al. demonstrated that patients with a high degree of myopia have a six times greater chance of developing primary OAG^(5)^. Another study showed that for every one diopter increase in myopia, there is a 20% increase in the risk of glaucoma. This rises even more sharply from six diopters and up^(2)^. As the two conditions can coexist, detecting glaucomatous changes in highly myopic nerves presents a diagnostic challenge due to the difficulty distinguishing between the two pathologies.

## PREOPERATIVE CONSIDERATIONS

Before any refractive procedure, a thorough ophthalmological evaluation should be performed. This should include a complete examination of the anterior and posterior segment, with photographs of the optic nerve as baseline documentation, especially in patients at increased risk of glaucoma^(6)^. A recent epidemiological study found that 5.1% of myopic individuals seeking refractive surgery had undiagnosed glaucoma and none were aware of their diagnosis at presentation^(7)^.

### Intraocular pressure evaluation

IOP pressure is estimated by tonometry in pre-and postoperative refractive surgery evaluations of myopes. IOP has been found to influence lowand high-order aberrations, which are evaluated by an aberrometer. The Goldmann applanation tonometer (GAT) is the international gold standard. However, it has several limitations. The measurements obtained using this tonometer are affected by scleral stiffness and the thickness and biomechanical properties of the cornea. Measurements exclusively taken in a vertical position can also reduce accuracy. The IOPs of eyes with a corneal thickness <525 µm tend to be underestimated, while those with a corneal thickness >555 µm tend to be overestimated. Therefore, measurement of corneal thickness is recommended when evaluating IOP. Corneal irregularities in astigmatism greater than three diopters tend to cause errors in IOP measurements, leading to underestimation along the flatter axis. In these cases, 90° rotation of the tonometer is recommended to obtain at least two measurements^(8)^.

In patients with high ametropia, photoablation affects corneal thickness and ocular characteristics. This can cause difficulties in the diagnostic evaluation of patients undergoing refractive surgery. In recent years, several new devices have been developed that aim to provide more accurate measurements of IOP by correcting for corneal characteristics. These include the Pascal, ocular response analyzer (ORA), and Corvis ST, which have already been demonstrated to provide more accurate values.

The ORA and the Corvis ST are noncontact tonometers that evaluate corneal biomechanics and correct IOP based on these parameters^(9)^. Corneal hysteresis is constant throughout the day and is not associated with refractive error or axial length. The reproducibility of estimates from both the ORA and the Corvid ST is adequate. It should be noted that different tonometers perform differently depending on the ocular conditions and are not interchangeable. It is important to document IOP at different time points with the same tonometer and to obtain more than one measurement from each patient for diagnostic and follow-up purposes^(10)^.

The Pascal tonometer seems to be unaffected by changes in corneal biomechanics following refractive surgery, probably because of its ability to measure IOP transcorneally, without the need to applanate the corneal surface. This type of tonometer is a good choice for patients who have undergone refractive surgery^(9)^.

### Structural and anatomical assessment

To evaluate for anomalies and risk factors for elevated IOP and glaucoma development, biomicroscopy of the anterior segment is necessary. Conjunctival changes such as those induced by allergies may indicate a history of corticosteroid use. Corneal changes such as pigmentation on the posterior surface, iridescent atrophies, and the presence of nodules may indicate pigment dispersion syndrome or iridocorneal endothelial syndrome. Gonioscopy is essential in cases of peripheral convexity of the iris, appositional closure, and pigment deposition^(11)^.

Fundoscopy is essential to evaluate the optic disc region, the neural ring, and the peripapillary nerve fiber layer. This must be complemented with standard retinography and red-free images to obtain comparative parameters for future evaluations^(11)^. Quantitative assessment is achieved using optical coherence tomography (OCT). A tilted myopic optic disc with areas of peripapillary atrophy limits the assessment of the neural ring in suspected glaucoma. However, this can be adequately measured by OCT, which can define the limits of Bruch’s membrane^(12)^. In highly myopic patients, losses in the fiber layer tend to affect the central region more. Such losses tend to be more peripheral in those with less severe myopia.

Myopic eyes often present with anatomical changes in the shape and size of the optic disc, as well as peripapillary atrophy. Therefore, the comparison of the same eyes at different time points can be fundamental in establishing a diagnosis. Eyes with myopic changes in the posterior pole present with thinning of the superior and inferior nerve fiber layer and of the ganglion cell complex in the macular area. This causes anatomical distortion, with spreading and tilting of the fibers and vessels toward the macular area. When the device compares this to the parameters of a normal eye, it can lead to the detection of defects or changes in normal individuals, causing diagnostic errors^(3)^.

In candidates for refractive surgery, functional assessment with perimetry may be limited to those with or suspected glaucoma. However, documentation of the structure of the optic nerve head and peripapillary nerve fiber layer is recommended. In eyes with progressive myopia, there may be an increase in beta-type peripapillary atrophy and torsion of the optic disc. These changes can lead to the appearance of scotomas, which suggests the progression of the perimetric defect and requires a differential diagnosis with glaucoma.

### Functional assessment

Whenever possible, perimetry should be used for visual field assessment in patients whose ametropia is corrected using contact lenses. The risk of artifacts related to the edge of the corrective lens is minimized using this method. Glaucoma and suspected glaucoma patients should undergo baseline visual field testing prior to refractive surgery. It can be difficult to accurately assess both disc appearance and the retinal nerve fiber layer in highly myopic eyes^(10)^. Therefore, perimetry provides a valuable functional test when evaluating eyes before and after laser correction. As peripheral points may be particularly affected by lens and artifact testing in myopes, care should be taken when assessing changes in perimeter performance.

Larger optic discs with more extensive peripapillary atrophy have larger blind spots and temporal scotomas. In early glaucoma, these defects appear more in the periphery in those with low myopia; while, in those with low myopia, they are more central^(13)^.

In eyes with progressive myopia, there may be an increase in the area of beta-type peripapillary atrophy and torsion of the optic disc. These changes can lead to the appearance of scotomas, which suggests the progression of the perimetric defect and requires diffe-rential diagnosis with glaucoma^(14)^.

Refractive procedures do not cause consistent changes in the visual field; however, highly myopic patients sometimes show perimetric changes, such as an increase in the blind spot and temporal scotoma. These are usually related to the anatomical peculiarities in these eyes (i.e., disc tilt and extensive peripapillary atrophy). These defects may worsen over time without necessarily being related to glaucoma.

## INTRAOPERATIVE COMPLICATIONS

Theoretically, transient increases in intraoperative IOP can induce glaucomatous nerve damage. IOP can increase to levels exceeding 65 mmHg during laser-assis-ted in situ keratomileusis (LASIK) flap creation using a microkeratome^(15)^.

For patients with glaucoma or suspected glaucoma who have already undergone refractive surgery, new tonometry methods and improved knowledge of the anatomical and functional variations in high myopia are needed for accurate diagnosis and adequate monitoring. When corrective surgery is indicated in such patients, a conservative approach may be safer. To establish whether this is the case, anatomical and functional documentation, careful assessment of the presence of glaucoma, staging, and long-term control of the disease, based on IOP and the stability of serial examinations are all vital.

## POSTOPERATIVE INTRAOCULAR PRESSURE MONITORING

Following refractive surgery, several IOP-related issues can arise in myopic patients. First, the reliability of tonometry becomes more complex because different tonometers work well with different corneal thicknesses^(11)^. Second, high myopia has been reported as a risk factor for a more pronounced steroid response. Therefore, there is a risk of steroid-induced ocular hypertension as the postoperative medical regimen usually includes topical steroids. Finally, while possible postoperative IOP spikes would probably not damage the optic nerve in a healthy eye, eyes with high myopia and the corresponding increase in axial length are more biomechanically vulnerable to IOP-induced damage^(16)^. Therefore, these patients require close postoperative IOP monitoring.

Patients who have undergone corneal refractive surgery are susceptible to interface fluid syndrome (IFS), also known as pressure-induced stromal keratopathy^(17)^, due to increased IOP and/or corneal endothelial dysfunction^(18)^.

In IFS, the presence of fluid under the corneal stromal flap reduces the effective surface contacting the applicator, resulting in artificially low-pressure readings by GAT^(19)^. Undetected increases in IOP in patients with undiagnosed IFS can have devastating consequences, including ischemic optic neuropathy^(20)^. Rebound tonometry and Corvis ST tonometry^(21)^ may be more accurate in patients with IFS because the IOP is not estimated until the probe’s movement is halted by the firmer posterior surface of the cornea.

Table 1 shows studies that have compared changes in the mean central cornea thickness and the mean IOP after refractive surgery with different tonometers. It seems that the Pascal tonometer is least affected by the corneal biomechanical changes following refractive surgery, probably because it does not need to applanate the corneal surface^(22)^.

**Table 1:** Assessment of the mean central corneal thickness and intraocular pressure changes after refractive surgery for glaucoma using different tonometry devices

Authors/Year	Number of patients	Mean CCT change	Mean IOP GAT change	Mean device adjusted IOP change	Alternative tonometer device
Iglesias et al., 2022(^9^)	30	-74	-2.76	-2.83	ORA
Soiberman et al., 2012(^23^)	30	-86.26	-5.53	-0.09	Pascal
Aristeidou et al., 2010(^15^)	182	-71.7	-3.70	NA	Pascal
Siganos et al., 2003(^24^)	60	-78.0	-5.40	NA	Pascal
Ang et al., 2022 (^10^)	60	-67.7	-3.77	-1.88	Corvis ST

CCT= central corneal thickness; GAT= Goldmann applanation tonometer; IOP= intraocular pressure; NA= not available; ORA= ocular response analyzer.

## MANAGING GLAUCOMA AND SUSPECTED GLAUCOMA PATIENTS AFTER REFRACTIVE SURGERY

In patients who have already undergone refractive surgery, certain factors can make the diagnosis of glaucoma more difficult. These include the effects of photoablation on corneal thickness, and ocular characteristics specific to some patients (primarily those with high myopia).

Accurate assessment of IOP is probably the most challenging part of the glaucoma workup with patients who have already undergone refractive surgery. Using GAT (the method most frequently used in clinical practice), we often lose the pressure reference in operated eyes, resulting in underestimated IOP measurements. One study found the progression of glaucoma to be greater in myopic eyes that have undergone refractive surgery than in those that have not^(19)^. This is likely due to the underestimation of IOP and suboptimal treatment in these cases. The assessment of glaucoma in myopic eyes requires a multifactorial approach. It is expected that, with improvements in relevant technology, we will be able to identify and define glaucomatous damage in myopic discs, providing a deeper understanding of the disease.

Although glaucoma is a relative contraindication for refractive surgery, the procedure can be safe for many patients with adequate perioperative management and follow-up. Advances in diagnostic modalities have facilitated earlier detection of the disease and subsequent earlier intervention when indicated^(5)^. However, there have been few studies on the subject, and there are no guidelines that clearly indicate the best way to manage these cases. In patients with glaucoma or suspected glaucoma, our recommendations differ depending on whether they have already undergone refractive surgery. In those who have already had the surgery, new tonometry methods (adjusted for biomechanical characteristics of the cornea) and improved knowledge of the anatomical and functional variations in high myopia are needed for more accurate diagnosis and adequate follow-up of these patients^(11)^. In those for whom the surgery is being considered, we believe that a more conservative approach is safer, especially in the absence of definitive guidelines. We must also reiterate the importance of anatomical and functional documentation in all candidates for refractive surgery. We also recommend careful assessment for the presence of glaucoma, staging, and long-term control of the disease. This should be based not only on IOP but also on the stability of the findings from serial examinations.

Finally, we hope that this article can help clinicians with decision-making in daily practice. We look forward to new studies, tools, and techniques that contribute to better management of these cases.
